# Technical report: clinical feasibility of augmented reality-navigated chronic subdural hematoma evacuation

**DOI:** 10.1093/jscr/rjae341

**Published:** 2024-05-28

**Authors:** Joshua Olexa, Annie Trang, Timothy Chryssikos, Gary Schwartzbauer, Bizhan Aarabi

**Affiliations:** Department of Neurosurgery, University of Maryland School of Medicine, 22 S. Greene Street, S12D, Baltimore, MD 21201, United States; Department of Neurosurgery, University of Maryland School of Medicine, 22 S. Greene Street, S12D, Baltimore, MD 21201, United States; Department of Neurosurgery, University of Maryland School of Medicine, 22 S. Greene Street, S12D, Baltimore, MD 21201, United States; Department of Neurosurgery, University of Maryland School of Medicine, 22 S. Greene Street, S12D, Baltimore, MD 21201, United States; Department of Neurosurgery, University of Maryland School of Medicine, 22 S. Greene Street, S12D, Baltimore, MD 21201, United States

**Keywords:** sugmented reality, chronic subdural hematoma, neurosurgery, trauma, navigation

## Abstract

Management of chronic subdural hematoma (cSDH) poses unique challenges and can be fraught with complications. Understanding the spatial relationships of cSDH to adjacent brain tissue and skull topography is critical for successful surgical treatment. The aim of this report is to highlight the feasibility and efficacy of a novel augmented reality (AR) overlay tool for surgical planning with technical description of two surgical cases using AR for surgical management of cSDH. This report describes a fiducial-less AR system for surgical planning of surgical evacuation of cSDH. The AR system was used to superimpose 3D anatomy onto the patients head to provide image guidance during two cases of evacuation. Imaging demonstrated convexity cSDH. A 3D model of the patient’s anatomy was created and registered onto the patients’ heads using a novel AR system. Surgical evacuation of the cSDH was completed in each case with surgical planning assisted by AR overlay.

## Introduction

Chronic subdural hematoma (cSDH) can pose challenges for surgeons due to various anatomical considerations. cSDH is estimated to be the most common pathology treated by neurosurgeons by 2050 [1]. Successful surgical intervention for cSDH necessitates an understanding of the adjacent brain anatomy that is likely affected and distorted by this mass hemorrhagic lesion, as well as the relationship of the mass lesion to skull topography. Often, the proximity of eloquent brain regions, fragile blood vessels, and the potential involvement of multiple lobes create a surgical scenario that can benefit from enhanced precision [2, 3]. Subdural hematoma evacuations are often performed without intraoperative image guidance, likely due to setup time, cost of traditional navigation, and various institutional logistics. Surgeons typically rely on the patient’s pre-operative computed tomography (CT) and/or magnetic resonance imaging (MRI) imaging with or without pre-operative fiducial placement [4].

While this is often sufficient for most cases in which a large craniotomy is completed, issues of limited exposure or ‘over-exposure’ leading to injury of otherwise unaffected brain regions can occur [5, 6]. Absence of intraoperative navigation can also increase the risk, while rare, of wrong sided surgery [7]. Given this need for a fast and efficient navigation system to be used in a trauma setting, we sought to explore alternative planning solutions which provide neurosurgeons with image-guidance options while fitting seamlessly into the operative workflow for cSDH surgery.

Augmented reality (AR) has emerged as a transformative technology in the field of neurosurgery, offering advancements in speed, visualization and guidance during procedures [8, 9]. In this specific clinical context, AR represents a promising option that can potentially improve safety and precision without disrupting standard surgical workflows. By overlaying digital representations of normal and pathological anatomy onto the surgeon’s real-time view of the patient’s anatomy, AR can enhance surgical navigation and decision-making [10]. Here we report on two cases in which AR was used as an image guidance solution during surgical planning for cSDH evacuation.

### Augmented reality technology platform

The AR system uses fiducial-less system for rapid image registration (Hoth Intelligence, Philadelphia, Pennsylvania). The system uses surface landmarks and computer vision detection of facial surfaces to overlay a 3D anatomic model—generated from patient Digital Imaging and Communications in Medicine (DICOM)—onto the patients’ head when viewed through the Microsoft Hololens 2 headset [11]. The system uses sensors built in to the Hololens 2 headset, and therefore the entire image guidance platform operates solely out of the headset.

### Registration time

For the two cases described in this report, the average registration time was 11.9±2.1 s.

### Augmented reality intraoperative planning

When the surgeon looks at the patients’ face, the model is registered and displayed on the patients’ head. The models generated for each patient consisted of six layers including face (gray), hematoma (blue), vasculature (red), brain (orange), skull (white), and ventricles (yellow). Once registered with the head, the surgeon can hide, show, or change the transparency of individual anatomic layers. Additionally, the system allows the surgeon to place markings and trajectories using a virtual marking pen. As such the surgeon not only can visualize critical anatomic structures but can use that visualization to map the location of a planned burr hole and/or craniotomy.

### Ethical approval

IRB approval for application of this technology was obtained via the Institutions’ IRB committee and allowed for intraoperative use for presurgical planning in the setting of this technology. The patient consented to the procedure and the participants and any identifiable individuals consented to publication of his/her image.

## Cases

### Case 1

**Figure 1:**
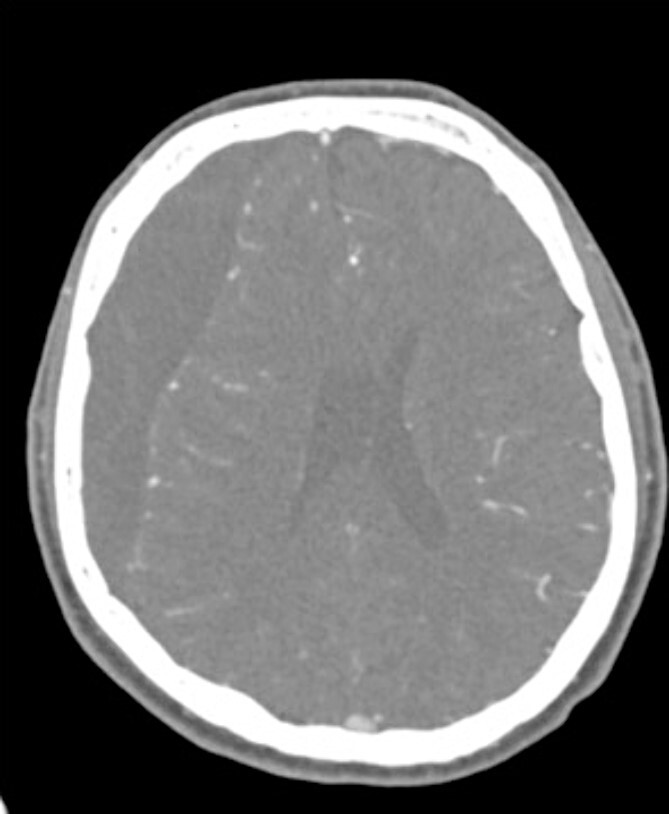
Preoperative CT imaging from Case 1.

A 60-year-old male with cSDH presented to a Level I trauma center with lethargy, headaches, and left-sided hemiparesis. CT demonstrated thickened membranes and midline shift (Fig. 1).

#### Surgical procedure with AR overlay

**Figure 2:**
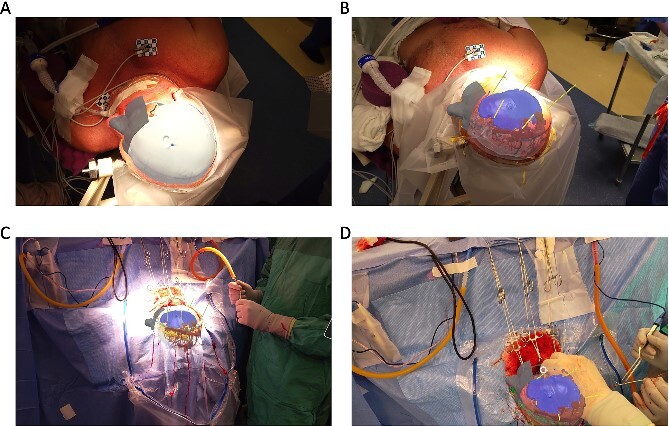
(A, B) Surgeons view through the AR headset displaying the 3D model overlaid on the patient from Case 1 preoperatively and (C, D) intraoperatively showing vasculature, hematoma, ventricles, and skull. Virtual trajectories are placed by surgeon to indicate a planned burr hole location.

**Figure 3:**
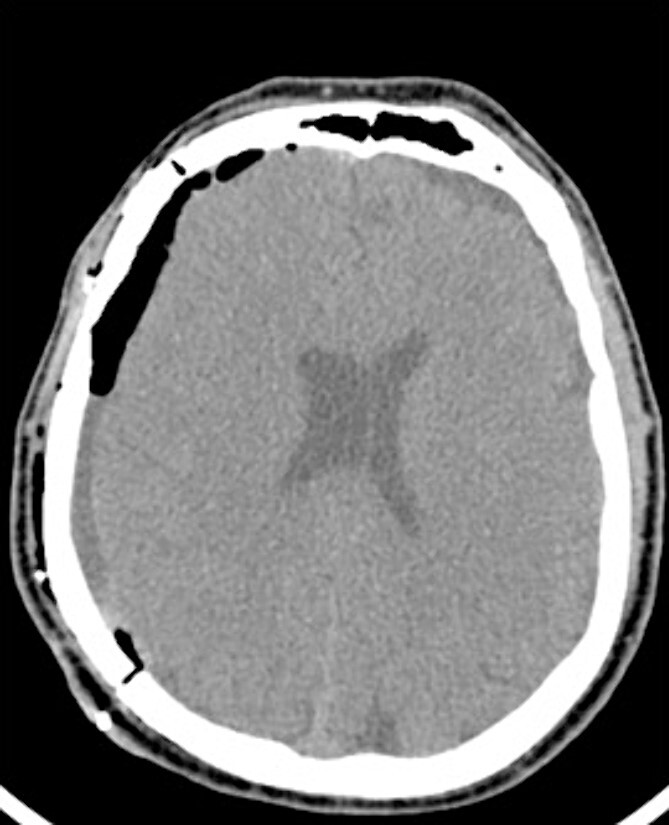
Postoperative CT imaging from Case 1.

An AR system was used to register the patient’s 3D anatomy onto the patients’ head (Fig. 2). Burr hole locations were annotated onto the registered 3D model to assist with craniotomy planning. Visualization of the 3D model confirmed plans for safe burr hole placement away from the dural venous and bony sinuses for a right frontoparietotemporal craniotomy. The AR headset was used once again to visualize the hematoma, vascular structures, and to confirm proper burr hole locations. The evacuation of the subdural hematoma was performed. Eight burr holes were placed, and a 14 × 11 cm bone flap was elevated to access the dura. The dura was then opened in a curvilinear fashion and hinged over the superior sagittal sinus. The cSDH was identified by its thick parietal and visceral membranes and underlying liquefaction, and these anatomic layers correlated directly with the AR overlay. The membranes of the hematoma were resected with the cortex demonstrating no gross abnormalities. The wound was irrigated, and the dura was closed primarily. The bone flap was then secured with two central tack-up structures followed by titanium reconstruction with multiple burr hole covers. There were no intraoperative complications, and the patient tolerated the procedure well with improved strength and decreased headache postoperatively. Post-operative CT showed interval decrease in the mixed-density subdural collection and reduced midline shift (Fig. 3).

### Case 2

**Figure 4:**
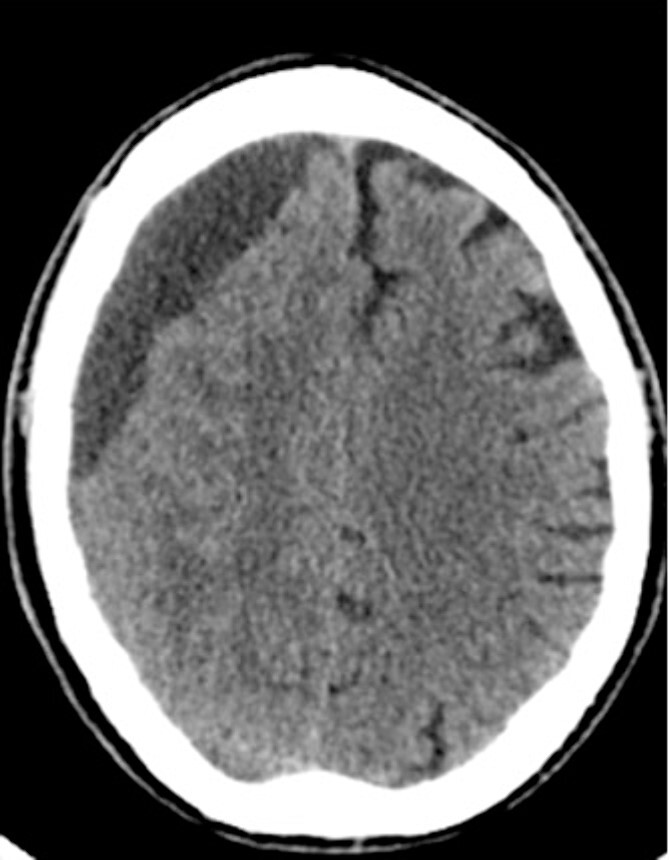
Preoperative CT imaging from Case 2.

An 81-year-old male with a history of acute subdural hematoma, previously managed non-surgically, presented with worsened confusion and cognitive dysfunction. Repeat CT demonstrated expansion of right-sided subdural collection, consistent with cSDH (Fig. 4).

#### Surgical procedure with AR overlay

**Figure 5:**
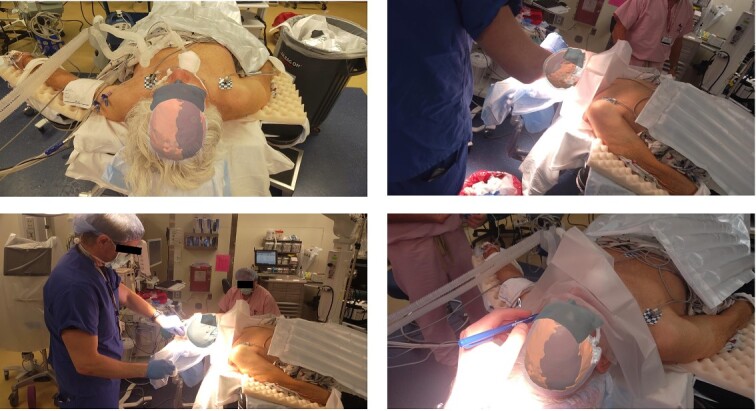
Surgeon’s view through the headset displaying the 3D model registered into the patients’ head from Case 2 showing hematoma, brain, skull, ventricles. Surgeon used AR display to mark incision and craniotomy location.

**Figure 6:**
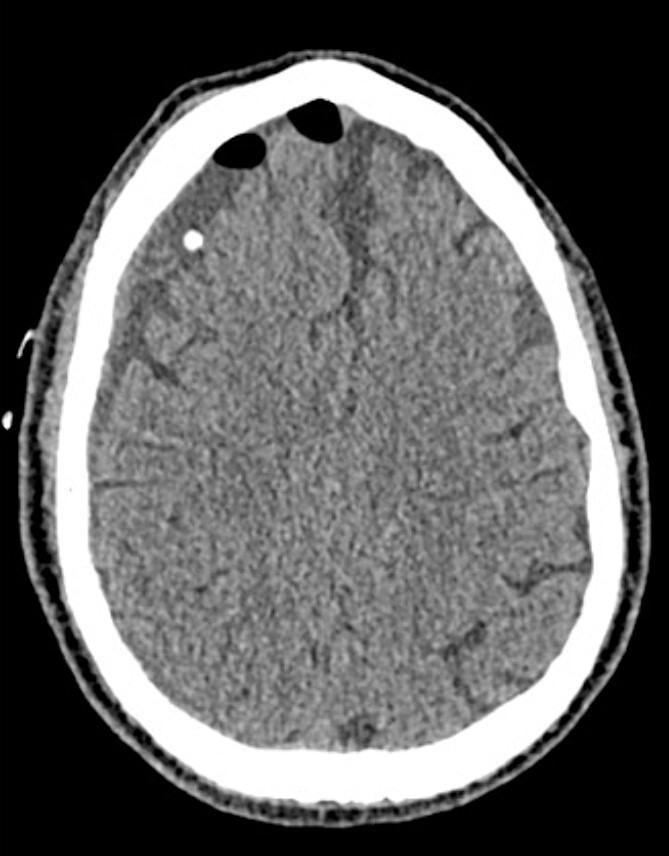
Postoperative CT imaging from Case 2.

After the head was positioned in a supine neutral position on a horseshoe head holder, the AR system was used to overlay the hematoma and other relevant anatomy onto the patient’s head. Midline locations, vasculature and the blood collection were appreciated via AR overlay prior to marking an incision (Fig. 5). The AR overlay was then used to plan two separate linear incisions for two right-sided burr holes—one frontal and one parietal over the largest components of the hematoma as visualized with AR. A perforator drill was used to create the two burr holes which were widened with a burr and rongeurs. Beneath the dura, a hematoma was identified and its thick membrane layer was coagulated with bipolar cautery. The surgical sites were irrigated with saline to evacuate chronic blood. A ventriculostomy catheter was then placed into the subdural space at the frontal burr hole and tunneled lateral to the incisions. The burr holes were covered with titanium covers. There were no intraoperative complications, and the tolerated procedure well with improved cognitive function several days after surgery. Post-operative CT demonstrated significant interval decrease in hematoma volume and reduction in midline shift (Fig. 6).

## Discussion

This technical report demonstrates the safety and feasibility of AR assistance for intraoperative surgical planning and guidance for evacuation of cSDH. Although this is a logical application of AR, to our knowledge, this is the first clinical report describing the use of AR for cSDH surgery. Currently, a number of neurosurgical procedures including bedside cranial procedures (e.g. EVD placement, ICP monitor placement, subdural drain placement, SEPS drain placement, etc.), emergency craniotomies and decompressive craniectomies, and ventricular shunt procedures are not performed with image guidance due to long setup times, cost, lack of availability, and/or cumbersome nature of the technology which may outweigh the benefit of image guidance [12]. Technological alternatives which provide enhanced visualization without the limitations of traditional navigation systems are needed. Here we describe a rapid, small footprint, and portable AR system that fits well into the surgical workflow and provides the surgeon with image guidance during cSDH evacuation.

The AR system successfully registered patient-specific 3D anatomy in two surgical cases onto the patient’s head and allowed the surgeon to precisely visualize the location of the hematoma in relation critical structures including bony sinuses, blood vessels, and underlying brain tissue. Unlike traditional navigation systems which can require long setup times and have a large OR footprint, the AR system registers 3D anatomy in approximately eleven seconds and has a small OR footprint (the system is entirely within a 1.2 lb headset worn by the surgeon). The option to plan and mark burr hole locations with the hematoma in view may increase procedural efficiency and safety [13].

While the potential of this technology is evident, the study has limitations that will be addressed in future work. This is a technical report including a small retrospective case series of two cSDH patients demonstrating the feasibility, potential merits, and limitations of AR overlay technology. Expanding use of this technology will provide greater clarity for its advantages and shortcomings. Nevertheless, the potential of this technology to improve safety and precision during cSDH evacuation is promising to formally compare AR-assisted evacuation to unnavigated surgery, clinical data collection including procedural speed, evacuation success, and other radiographic and patient outcomes are warranted. This may be especially valuable for junior neurosurgical trainees with limited experience, as well as community and military settings that may lack sufficient neurosurgical personnel or equipment.

## Conclusions

Augmented Reality is as a promising technology in neurosurgery. While the technology is still in its infancy, there are neurosurgical procedures that can leverage the speed and size of AR systems to provide surgeons with image guidance solutions for enhanced surgical precision. Two cases of cSDH evacuation using AR overlay for preoperative and intraoperative image guidance demonstrate the safety and feasibility of this modality. The system offers a promising image-based supplement to traditionally non-navigated procedures and may increase the safety and efficiency of cSDH evacuation and other surgical procedures.
